# Heparin-binding protein in sputum as a marker of pulmonary inflammation, lung function, and bacterial load in children with cystic fibrosis

**DOI:** 10.1186/s12890-018-0668-7

**Published:** 2018-06-20

**Authors:** Gisela Hovold, Victoria Palmcrantz, Fredrik Kahn, Arne Egesten, Lisa I. Påhlman

**Affiliations:** 1 Department of Clinical Sciences Lund, Division of Infection Medicine, BMC B14, Lund University, Skåne University Hospital, Tornavägen 10, SE-22184 Lund, Sweden; 2 Skåne University Hospital, Clinic of Paediatrics, Lund, Sweden; 3 Department of Clinical Sciences Lund, Respiratory Medicine & Allergology, Lund University, Lund, Sweden

**Keywords:** Cystic fibrosis, Sputum, Inflammation, Airway infection, Lung function, Children

## Abstract

**Background:**

Cystic fibrosis (CF) is associated with bacterial pulmonary infections and neutrophil-dominated inflammation in the airways. The aim of this study was to evaluate the neutrophil-derived protein Heparin-binding protein (HBP) as a potential sputum marker of airway inflammation and bacterial load.

**Methods:**

Nineteen CF patients, aged 6–18 years, were prospectively followed for 6 months with sputum sampling at every visit to the CF clinic. A total of 41 sputum samples were collected. Sputum-HBP was analysed with ELISA, neutrophil elastase activity with a chromogenic assay, and total bacterial load with RT-PCR of the 16 s rDNA gene. Data were compared to lung function parameters and airway symptoms.

**Results:**

HBP and elastase correlated to a decrease in FEV_1_%predicted compared to the patients´ individual baseline pulmonary function (∆FEV_1_), but not to bacterial load. Area under the receiver operating characteristic curve values for the detection of > 10% decrease in ∆FEV_1_ were 0.80 for HBP, 0.78 for elastase, and 0.54 for bacterial load.

**Conclusions:**

Sputum HBP is a promising marker of airway inflammation and pulmonary function in children with CF.

## Background

Cystic fibrosis (CF) is associated with persistent bacterial infection and neutrophil-dominated inflammation in the respiratory tract [1]. Several bacterial species cause airway infection in CF, for example *Staphylococcus aureus,* Non-typable *Haemophilus influenzae* (Nt*Hi*), *Burkholderia cepacia complex,* and *Pseudomonas aeruginosa,* and it is well described that exacerbations contribute to the progressive lung destruction and pulmonary function decline that is a hallmark of CF [2, 3]*.* Many bacterial species show increasing resistance to antibiotics, and there is therefore a need for robust biomarkers to monitor airway inflammation and infection. Such biomarkers could prevent unnecessary use of antibiotics that promote development of bacterial resistance.

Heparin-binding protein (HBP) is stored in the secretory and azurophilic granules of neutrophils, and is released upon cell activation [4]. HBP is a multi-functional pro-inflammatory mediator that for example activates immune cells, has broad antibacterial activity, and induces vascular leakage [5, 6]. Plasma HBP has been described as a promising predictor of the progression into severe sepsis and septic shock [7]. HBP levels also increase in cerebrospinal fluid during bacterial meningitis [8] and in urine during urinary tract infection [9], suggesting its use as a biomarker indicating bacterial infection. Recently, HBP was described as a useful biomarker in broncho-alveolar lavage fluid for the detection of pulmonary infection in lung transplant recipients [10].

The aim of the present study was to evaluate if HBP levels in sputum from children with CF can be used as a biomarker for pulmonary inflammation and bacterial load. Neutrophil elastase was analysed for comparison and as a marker reflecting neutrophil load in the airways.

## Methods

### Study population

CF patients, 6 to 18 years of age, at the paediatric CF centre of Skåne University Hospital in Lund, were eligible for inclusion in the study. Patients under shared care with other clinics were excluded. At each visit to the clinic, the subjects donated an expectorated or induced sputum sample to the study, and their lung function was evaluated with spirometry. Sputum was induced by administering individually tailored hypertonic saline (median 5%, range 3–7%), followed by airway clearance techniques such as positive expiratory pressure (PEP) and forced expiration technique (FET). Sputum was collected during or directly after airway clearance. The procedure was supervised by a physiotherapist. Forced expiratory volume in one second in % of predicted (FEV1%predicted) and forced expiratory flow between 25 and 75% of expired forced vital capacity in % of predicted (FEF%predicted) were determined from predicted values according to Zapletal [11]. A mean of the two best values of FEV_1_%predicted or FEF%predicted from the previous year were used as baseline to calculate the change in FEV_1_%predicted (∆FEV_1_) or change in FEF%predicted (∆FEF), respectively, at the time of sampling. At start of *iv* antibiotic treatment, a blood sample was also donated to the study.

Sputum cultures were analysed at the department of Medical Microbiology, Laboratory medicine, Skåne University Hospital, according to approved conventional methods in the routine laboratory [12]. Information about pulmonary symptoms such as dyspnoea, cough, fever, increased mucus, or change in sputum colour, was extracted from the medical journal. The sample collection was performed from January to June 2015.

A written informed consent was obtained from all participants and/or their guardian. The study was approved by the Medical Ethic Committee (Institutional Review Board) of Lund University (reference number 2011/434).

### Sample preparation

All sputum samples were taken care of within two hours of sampling. One aliquot of the sample was mixed with an equal volume of Saliva Preservation Solution (Norgen Biotek Corporation, Canada) and frozen at − 80 °C until further processing. The remaining portion of the sample was homogenized by mixing sputum 1:1 (*w*/*v*) with 0.1% dithiothreitol (DTT) in PBS. The samples were incubated at 37 °C for 20 min with intermittent mixing, followed by centrifugation at 3000 rpm for 10 min. The cell-free supernatants were collected and stored at − 80 °C until further analyses.

Blood samples were centrifuged at 3000 rpm for 10 min. Plasma was collected and stored at − 80 °C until analyses. All plasma samples were centrifuged and frozen within one hour from sampling.

### Quantification of HBP

HBP concentrations were analysed with ELISA as previously described [4]. All samples were assayed in duplicates.

### Neutrophil elastase activity

Ten μl of liquefied sputum sample was mixed with 90 μl of HEPES buffer (0.1 M HEPES, 0.5 M NaCl; pH 7.5), supplemented with 5 mM EDTA, 2 μg/ml of E64 (Sigma, St. Louis, MO) and 1 μg/ml of Pepstatin A (Sigma-Aldrich, St. Louis, MO) in a 96-wells plate. Neutrophil elastase (Sigma-Aldrich) in 2-fold dilutions from a 2 U/ml stem solution was used as a standard. Fifty μl of the chromogenic substrate N-methoxysuccinyl-Ala-Ala-Pro-Val p-nitroanilide (Sigma-Aldrich) was added to each well to a final concentration of 0.5 mM. The plates were incubated at 37 °C and the absorbance at 415 nm was determined every 10 min to measure enzyme kinetics. All samples were analysed in duplicates.

### Nucleic acid extraction and real-time PCR

Sputum samples were homogenized with DTT as described above. DNA extraction was performed with a Sputum DNA isolation kit (cat. #46200, Norgen Biotek Corporation) according to the manufacturer’s protocol.

Total bacterial load was quantified with real-time PCR of 16 s rDNA, using the following primer pair: forward: TGCCAGCAGCCGCGGTAA, reverse: AGGCCCGGGAACGTATTCAC. One μl of DNA template was added to 19 μl master mix containing SYBR® Green (cat. #172–5121, Bio-Rad, Hercules, CA), sterile water, and the forward and reverse primers. Expression was analyzed using the iTaq™ Universal SYBR® Green Supermix (Bio-Rad). Known amounts of *Staphylococcus aureus* DNA in 10-fold dilutions were analyzed in parallel and used as a standard. Amplification was performed at 54 °C for 35 cycles in an iCycler Thermal Cycler (Bio-Rad, Hercules, CA) and DNA concentrations were calculated from the standard curve.

### Statistics

Statistical calculations were done using the GraphPad Prism 7 software (GraphPad Software, San Diego, CA) and R (R Core Team 2017, R Foundation for Statistical Computing, Vienna, Austria) using R Studio (RStudio, Inc., Boston, MA) with installed packages nlme, mgcv, r2glmm, geepack and MuMln (https://cran.r-project.org/web/packages/r2glmm/index.html) [13, 14] (https://cran.r-project.org/web/packages/MuMIn/index.html). Comparisons between unpaired groups were made with the non-parametric Mann-Whitney U test, and with Wilcoxon matched-pairs signed rank test for paired observations. Correlations were done with Spearman rank coefficient. Cut-off values for the calculation of sensitivity, specificity and predictive values were chosen based on receiver operator characteristics data.

To account for possible dependency due to repeated measurements from the same patient, we used mixed model with random effects and generalised estimating equation (GEE) models with subjects as the random components. The linear relationship between biomarkers and lung function was confirmed using a mixed additive model with random effects. To assess the relative importance of each variable in the mixed model, a semi-partial r-square was calculated according to Jaeger et al. [15]. Two-tailed *P* < 0.05 and 95% confidence intervals (CIs) that did not overlap 1.0 were regarded as statistically significant.

## Results

### Patient characteristics

**Fig. 1:**
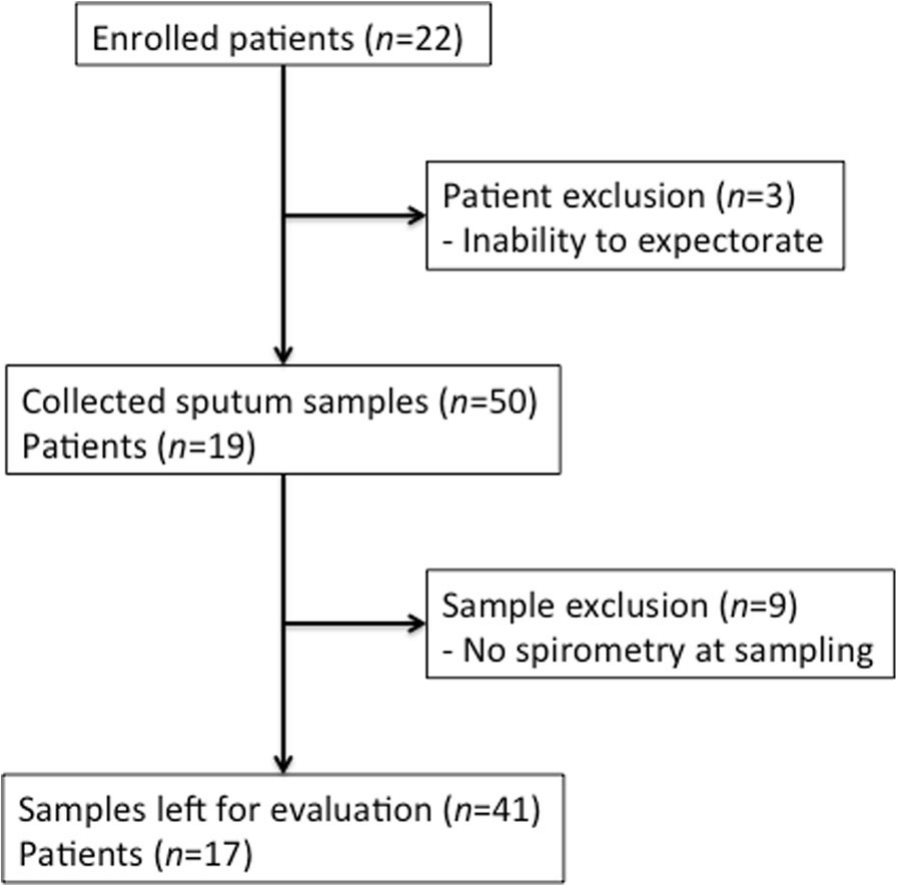
Flowchart of patients and sputum samples in the cohort

A total of 22 patients suffering from CF (aged 6–18 years) were eligible and enrolled in the study. The study participants had a median age of 9 years, and male sex was the dominating gender (Table 1). The participants were followed with consecutive sputum sampling during a six months period, from January to June 2015. Three patients were unable to donate a sputum sample during the study period and were therefore excluded (Fig. 1). The remaining 19 patients donated a total of 50 sputum samples. Nine sputum samples were excluded as no lung function evaluation had been performed at the time of sampling. This left 41 sputum samples from 17 patients, with a median of 2 samples per patient (range 1–4 samples). 83% of sputum samples were induced with sodium chloride. Samples collected from the same patient were obtained with a median of 37 days apart (range 7–176 days). Another 6 samples were excluded from DNA analyses, as they contained small volumes of sputum that only allowed HBP and elastase measurements.

**Table 1:** Patient characteristics

Median age (IQR)^a^	9 (8–11)
Male gender; % (*n*)	71 (12)
CFTR mutation; % (n)	
- ΔF508/ΔF508	59 (10)
- Others	41 (7)
Pancreas insufficiency; % (n)	100 (17)
No of samples/patient; median (IQR)	2 (2–3)
Induced sputum; % (*n*)	83 (34)
Pseudomonas colonization^b^; % (*n*)	
- Never infected	24 (4)
- Free of infection	6 (1)
- Intermittent infection	53 (9)
- Chronic infection	18 (3)
Sputum culture growth; % (*n*)	
- *Staphylococcus aureus*	34 (12)
- *Pseudomonas aeruginosa*	26 (10)
- *Haemophilus influenzae*	14 (5)
- *Burkholderia cepacia*	6 (2)
- Negative culture	6 (2)

^a^IQR = Interquartile range

^b^Pseudomonas aeruginosa (PA) colonization according to Leeds criteria: Never infected, Free of infection (no PA growth during the previous 12 months), Intermittent infection (PA growth in 50% or less of cultures), or Chronic infection (PA growth in > 50% of cultures)

*Staphylococcus aureus* and *Pseudomonas aeruginosa* were the most commonly found bacterial species in sputum cultures (32 and 24%, respectively). Thirty-six % of sputum samples were collected during on-going symptoms from the respiratory tract (dyspnoea, cough, fever, increased mucus, or change in sputum colour). Nine samples were collected at start of intravenous antibiotic treatment. Six of these treatments were initiated to eradicate *P. aeruginosa* and the remaining to treat exacerbations.

### HBP, elastase and bacterial load in sputum

**Fig. 2:**
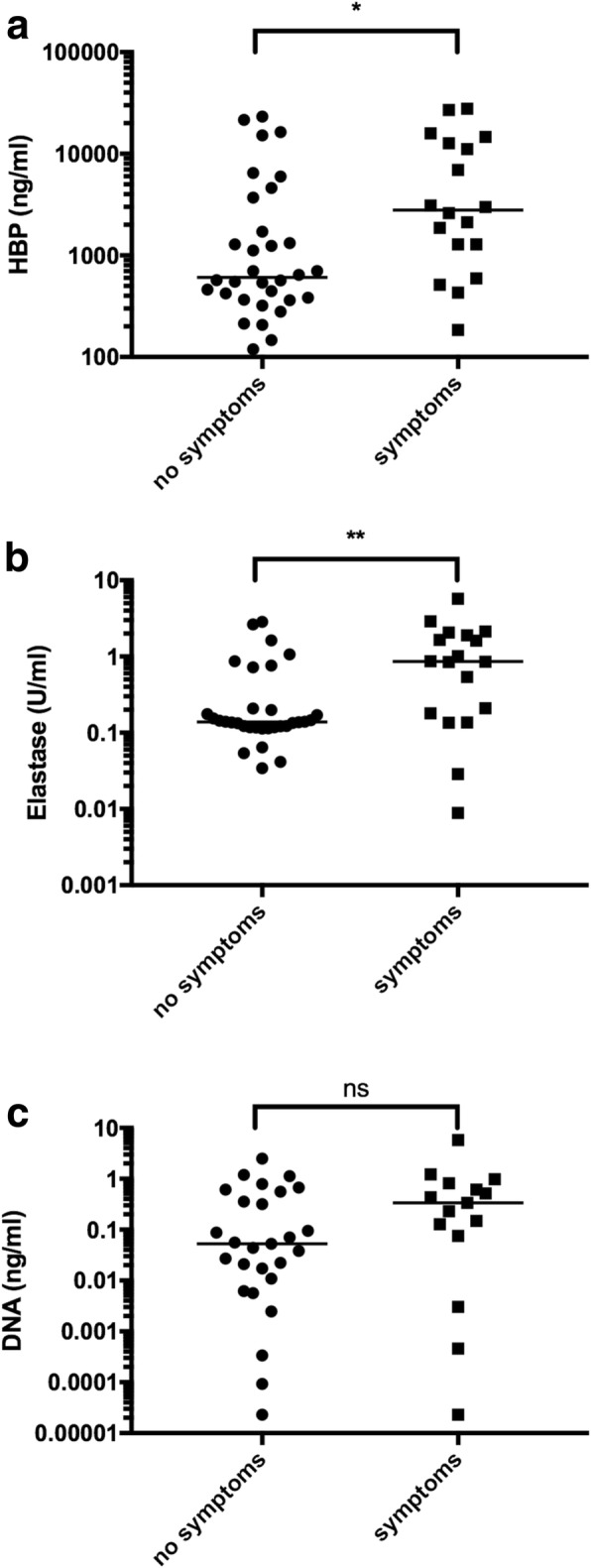
Sputum levels of HBP (**a**), elastase (**b**), and bacterial load (**c**) in patients with and without symptoms from the respiratory tract. * = *p* < 0.05. ** = *p* < 0.005. Ns = not significant

Sputum concentrations of HBP and elastase, but not bacterial load, were significantly increased in samples collected in the presence of respiratory symptoms (Fig. 2). To account for the possibility of bias due to repeated measures from the same patient, logistic regression with general estimating equation (GEE) models was performed using the logarithmic values of HBP, elastase and bacterial DNA as continuous variables. Using GEE analyses, the odds ratios (ORs) for the prediction of respiratory symptoms were 1.4829 (95% CI 1.02766–2.140) for log(HBP), 1.705 (95% CI 1.082–2.69) for log(Elastase), and 1.124 (95% CI 0.972–1.30) for log(bacterial load).

**Fig. 3:**
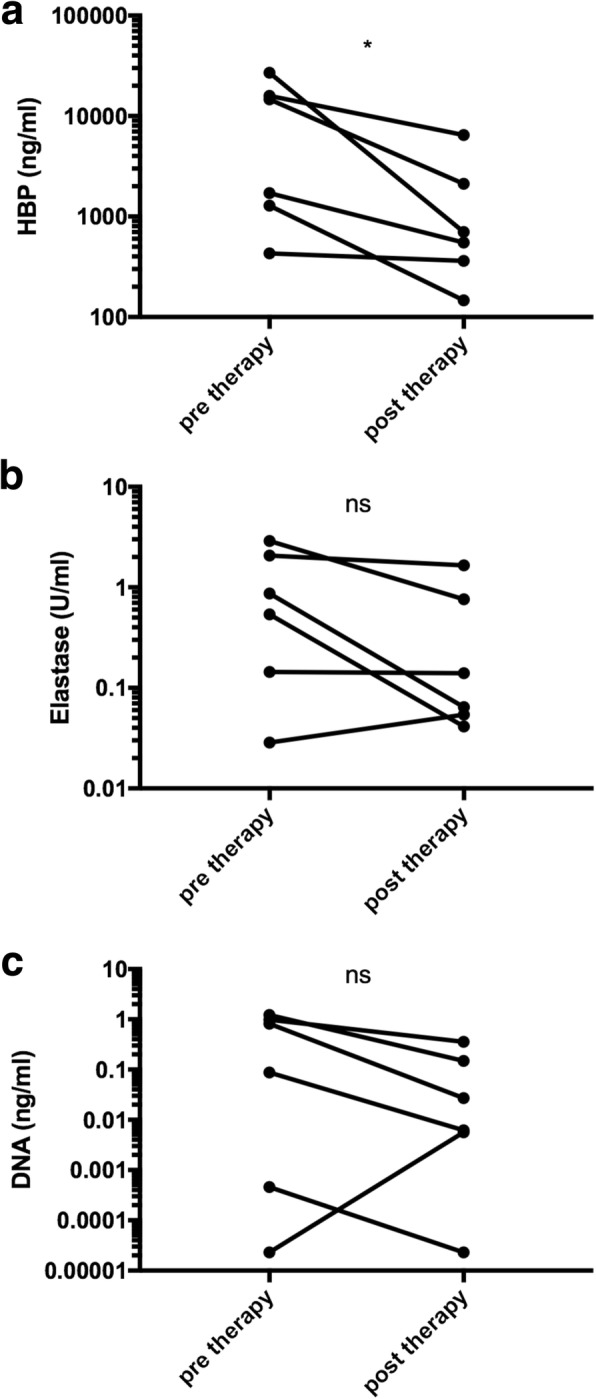
Levels of HBP (**a**), elastase (**b**), and bacterial load (**c**) before and after antibiotic treatment. * = *p* < 0.05. Ns = not significant

Six participants had a sputum sample taken at start of antibiotic therapy, and a follow-up sample taken within one month. Median values decreased after therapy for all markers, but only HBP reached statistical significance (Fig. 3). Neither levels of HBP nor elastase differed between induced and naturally expectorated sputum (*p* = 0.09 for HBP and *p* = 0.2 for elastase).

**Fig. 4:**
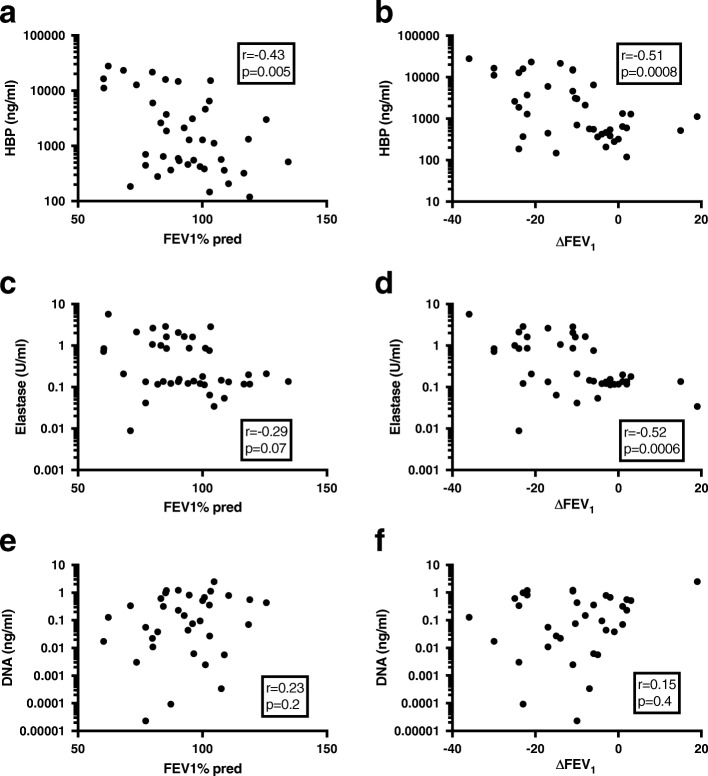
Correlations between biomarkers and lung function. HBP (**a** and **b**), elastase (**c** and **d**), and bacterial DNA (**e** and **f**) were correlated to FEV_1_%predicted or ∆FEV_1_. r = nonparametric Spearman rank coefficient

Next, HBP, elastase and bacterial DNA were correlated to FEV_1_%predicted at the time of sampling, and to the change in FEV_1_%predicted compared to baseline levels (∆FEV_1_). A mean of the patient’s two best FEV_1_%predicted values from the previous year was used as baseline, and the change in lung function was expressed in percent. Both HBP and elastase correlated more strongly to ∆FEV_1_ than FEV_1_%predicted, whereas no convincing correlation was found between total bacterial load in sputum and either FEV_1_%predicted or ∆FEV_1_ (Fig. 4). To account for repeated measurements from the same patient, the correlations between biomarkers and ∆FEV_1_ were validated both in a mixed model with patients as random effects, as well as using GEE. The mixed model with random effects estimated log(HBP) to − 2.5 (− 4.63 – − 0.379, *p* = 0.0229) and a semi-partial R-square of 0.122 (0.005–0.344). The estimate for log(elastase) was − 3.17 (− 5.41– − 0.933, *p* = 0.0075) with a semi-partial R-square of 0.161 (0.016–0.389), and for log(bacterial load) the estimate was 0.11 (− 1.23–1.44, *p* = 0.8705) with a semi-partial R-square of 0.001 (0–0.142). Importantly, GEE gave point estimates for the prediction of a decrease in ∆FEV_1_ in agreement with the mixed models, but only HBP remained statistically significant. The estimates from the GEE were − 2.57 (*p* = 0.023) for log(HBP), − 2.96 (*p* = 0.086) for log(Elastase), and − 0.0538 (*p* = 0.93) for log(bacterial load).

No correlation was detected between HBP and bacterial DNA (*r* = 0.09, *p* = 0.56) or between elastase and bacterial DNA (*r* = 0.24, *p* = 0.13), whereas HBP and elastase correlated well (*r* = 0.85, *p* < 0.001). No differences in sputum HBP or elastase were found between the different groups of *Pseudomonas* colonisation according to Leeds criteria (data not shown).

In order to reflect involvement of small airway disease [16], sputum HBP levels were also correlated to FEF25/75 in % of predicted values (FEF%predicted), and to a change in FEF%predicted compared to baseline levels (∆FEF%). HBP showed a weaker, but not statistically different correlation to both FEF%predicted (*r* = − 0.27, *p* = 0.09) compared to FEV_1_%predicted (*p* = 0.43), and to ∆FEF% (*r* = − 0.17, *p* = 0.30) compared to ∆FEV_1_, (*p* = 0.09).

**Fig. 5:**
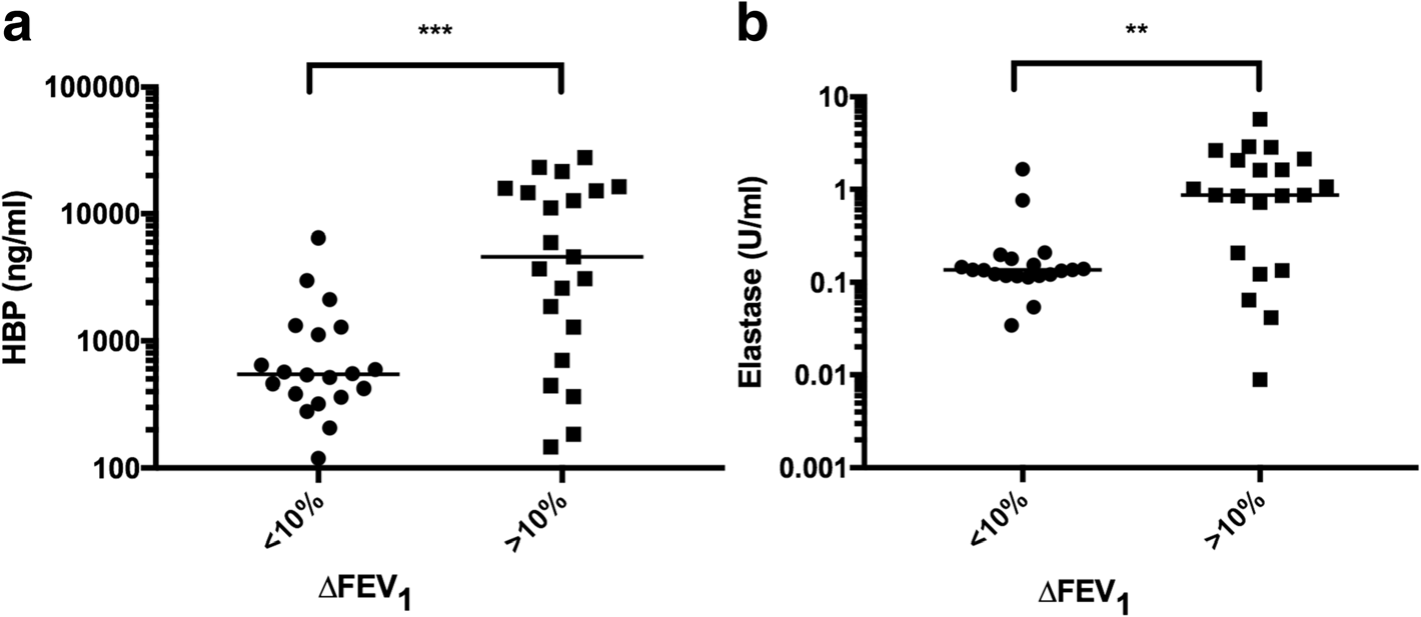
Sputum levels of HBP (**a**) and elastase (**b**) depending on ∆FEV_1_. Sputum samples with more than 10% decline in the patient’s ∆FEV_1_ were compared to samples with less than 10% decline in lung function. ∆FEV_1_ was calculated using the mean of the patient’s two best FEV_1_%predicted values the previous year as baseline, and comparing to the patient’s FEV_1_%predicted at the time of sampling, expressed in %. ** = *p* < 0.005. *** = *p* < 0.001

**Fig. 6:**
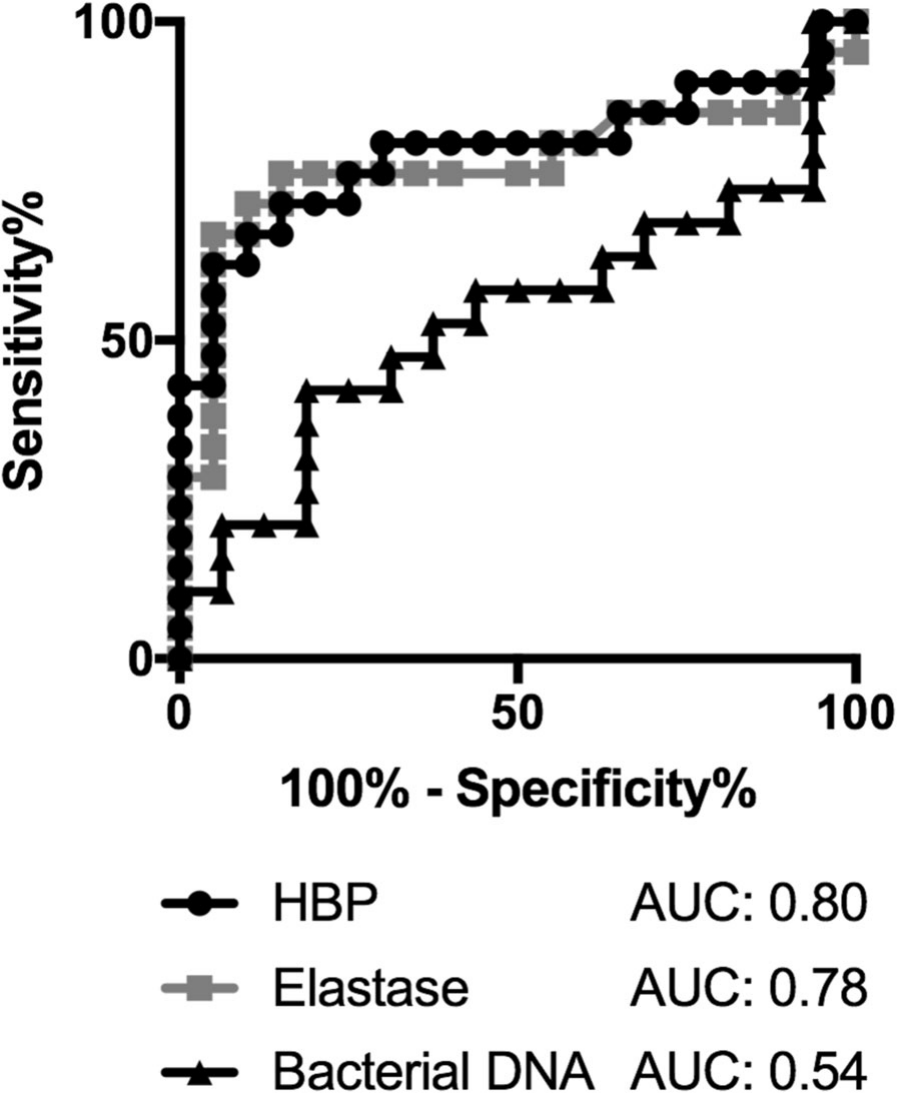
Receiver-operating characteristics (ROC) curves of HBP, elastase, and bacterial DNA for the detection of > 10% decline in ∆FEV_1_

Using 10% decline in ∆FEV_1_ compared to baseline as cut-off, both HBP and elastase were significantly increased in the group with a decrease in ∆FEV_1_ (Fig. 5). The levels of bacterial DNA were similar in both groups (data not shown). Receiver Operator Characteristics (ROC) analyses for the detection of > 10% decline in ∆FEV_1_ are shown in Fig. 6. HBP and elastase performed equally well, with area under the curve (AUC) values of 0.80 (95% CI 0.65–0.94) for HBP and 0.78 (95% CI 0.62–0.93) for elastase. Bacterial DNA presented a lower AUC of 0.54 (95% CI 0.34–0.73) for the detection of a decrease in ∆FEV_1_. Based on ROC data, cut-off values for the calculation of sensitivity, specificity and predictive values were identified for HBP, elastase and bacterial DNA. At a cut-off value of 650 ng/ml for HBP, the sensitivity was 81% and the specificity 70% for the detection of > 10% decline in ∆FEV_1_. The positive and negative predictive values were 74 and 78%, respectively. Elastase performed similar to HBP, whereas bacterial DNA showed poor sensitivity and specificity (Table 2).

**Table 2:** Sensitivity, specificity and predictive values for the detection of > 10% decrease in ∆FEV_1_

	Cut-off	Sensitivity (%)	Specificity (%)	PPV (%)	NPV (%)
HBP (ng/ml)	650	81	70	74	78
Elastase (U/ml)	0.15	76	70	73	74
Bacterial load (ng/ml)	0.1	47	44	50	41

*PPV* positive predictive value, *NPV* negative predictive value

### HBP levels in plasma

Eight patients donated a total of 9 plasma samples at the start of *iv* antibiotic treatment during the study period. The median HBP concentration in plasma was 7.0 ng/ml (range 5.1–20.5 ng/ml), which was significantly lower than the HBP concentrations in the corresponding sputum samples (median 1716 ng/ml, range 384–27,051 ng/ml, *p* < 0.01).

## Discussion

HBP has received attention over the last years as a promising biomarker of infection in sepsis, urinary tract infections, meningitis, and lately also as a marker of airway infection in broncho-alveolar lavage (BAL) of lung transplant recipients [7–10]. In this prospective study on children with cystic fibrosis, HBP levels in sputum correlated to lung function and to respiratory symptoms, but not to bacterial load. HBP levels in plasma were low and in the same range as reported in healthy individuals [7]. In comparison, levels of HBP in concomitant sputum samples were at least 100-fold and up to 1000-fold higher. The high concentrations of HBP in sputum underline the heavy neutrophil-dominated airway inflammation seen in CF patients, and probably reflect that the lung is the end organ where the neutrophil becomes activated and releases its granular contents.

Expectorated and induced sputum is a non-invasive method to obtain airway samples from CF-patients, as opposed to BAL which is still the gold standard for defining airway inflammation and microbiology in infants and young children [17]. Studies have shown that sputum samples are of equal value as BAL specimens to detect *P. aeruginosa* colonization [18], indicating that sputum biomarkers for the detection of airway inflammation and infection would be a simple and useful tool in clinical practice. However, sputum collection is not as standardized as BAL, which may result in large intra- and inter-individual variations. For example, CF patients often have sputum plugs that cannot be expectorated and thus cause false low levels of inflammatory markers. Moreover, the sampling technique was not identical for all samples in the study, as 17% of samples were expectorated and 83% were induced with sodium chloride. However, we saw no differences in biomarker levels between induced and non-induced sputum, and it has also been shown by others that there are no differences in microbiology between induced and expectorated sputum [19]. We therefore believe that the sampling technique has a minor impact on the results.

In this study, bacterial DNA tended to increase with pulmonary symptoms but did not reach statistical significance. Nor did bacterial DNA levels correlate to pulmonary function or biomarker levels. Previous work has reported conflicting results. For example, it has been shown that *P. aeruginosa* load correlate to lung function in stable patients, and also decrease after treatment of pulmonary exacerbation [20–22]. On the other hand, a large multicentre study could not attribute any important differences in FEV_1_ to bacterial densities [23]. In this study, the cohort was diverse with respect to airway microbiology. We therefore chose to analyse total bacterial load with a universal 16S rDNA primer, as not all participants were colonized with pseudomonas. This PCR-method detects both viable and dead cells, which may be one reason why not more clear results were obtained.

An important shortcoming of the study is the lack of information about sputum neutrophil counts. Instead, we used neutrophil elastase as a marker of the neutrophil burden in our comparisons with HBP. Neutrophil elastase is well studied in sputum from CF patients, and has been reported to decrease after treatment of pulmonary exacerbations [21], to have a longitudinal association with lung function [23], and to be predictive of lung function decline [24]. Both elastase and HBP are neutrophil granule proteins that are released upon cellular activation. It is therefore not surprising that HBP performed equally well to elastase in most, but not all, comparisons made in this study. For example, HBP decreased significantly in sputum after antibiotic treatment, whereas elastase did not. However, only 6 patients had sputum samples taken before and after treatment, which gives the comparison poor power. Moreover, HBP was the only biomarker that remained statistically significant in all comparisons regardless of statistical analysis.

Another difficulty is the definition of airway symptoms in the study. There is no universally accepted definition of pulmonary exacerbations in CF, although Fuchs criteria are commonly used for adults. However, it has been argued that Fuchs criteria are less suited for studies on children, who have a milder lung disease and also milder symptoms of exacerbation [25]. In this study, patients were simply classified based on symptoms from the lower respiratory tract as reported by the patient or staff. Only a few patients experienced exacerbations that required *iv* antibiotic treatment during the study period. Even so, HBP increased significantly in the presence of pulmonary symptoms.

A final limitation is the small number of patients and the fact that participants donated varying numbers of samples to the study. However, even after correction for multiple measurements with mixed models and GEE, we could demonstrate that HBP correlates to ∆FEV_1_ and pulmonary symptoms.

## Conclusion

Sputum HBP is a promising biomarker for pulmonary inflammation and lung function in children with CF, and could thus be a simple and complementary tool in clinical practice. However, more studies are needed on larger study populations to validate the results.

## Abbreviations

∆FEV_1_: Change in FEV_1_%predicted
BAL: Broncho-alveolar lavage
CF: Cystic Fibrosis
FEV_1_%predicted: Forced expiratory volume in one second, % of predicted
HBP: Heparin-binding protein
RT-PCR: Real-Time Polymerase Chain Reaction
